# Evidence-informed health policy 3 – Interviews with the directors of organizations that support the use of research evidence

**DOI:** 10.1186/1748-5908-3-55

**Published:** 2008-12-17

**Authors:** John N Lavis, Andrew D Oxman, Ray Moynihan, Elizabeth J Paulsen

**Affiliations:** 1 Centre for Health Economics and Policy Analysis, Department of Clinical Epidemiology and Biostatistics, McMaster University, 1200 Main St West, HSC-2D3, Hamilton, ON L8N 3Z5, Canada; 2 Department of Political Science, McMaster University, 1200 Main St West, HSC-2D3, Hamilton, ON L8N 3Z5, Canada; 3 Norwegian Knowledge Centre for the Health Services, Pb 7004, St Olavs plass, Oslo N-0130, Norway; 4 School of Medicine and Public Health, Faculty of Health, the University of Newcastle, Medical Sciences Building – Level 6, Callaghan, NSW 2308, Australia

## Abstract

**Background:**

Only a small number of previous efforts to describe the experiences of organizations that produce clinical practice guidelines (CPGs), undertake health technology assessments (HTAs), or directly support the use of research evidence in developing health policy (*i.e.*, government support units, or GSUs) have relied on interviews and then only with HTA agencies. Interviews offer the potential for capturing experiences in great depth, particularly the experiences of organizations that may be under-represented in surveys.

**Methods:**

We purposively sampled organizations from among those who completed a questionnaire in the first phase of our three-phase study, developed and piloted a semi-structured interview guide, and conducted the interviews by telephone, audio-taped them, and took notes simultaneously. Binary or categorical responses to more structured questions were counted when possible. Themes were identified from among responses to semi-structured questions using a constant comparative method of analysis. Illustrative quotations were identified to supplement the narrative description of the themes.

**Results:**

We interviewed the director (or his or her nominee) in 25 organizations, of which 12 were GSUs. Using rigorous methods that are systematic and transparent (sometimes shortened to 'being evidence-based') was the most commonly cited strength among all organizations. GSUs more consistently described their close links with policymakers as a strength, whereas organizations producing CPGs, HTAs, or both had conflicting viewpoints about such close links. With few exceptions, all types of organizations tended to focus largely on weaknesses in implementation, rather than strengths. The advice offered to those trying to establish similar organizations include: 1) collaborate with other organizations; 2) establish strong links with policymakers and stakeholders; 3) be independent and manage conflicts of interest; 4) build capacity; 5) use good methods and be transparent; 6) start small and address important questions; and 7) be attentive to implementation considerations. The advice offered to the World Health Organization (WHO) and other international organizations and networks was to foster collaborations across organizations.

**Conclusion:**

The findings from our interview study, the most broadly based of its kind, extend to both CPG-producing organizations and GSUs the applicability of the messages arising from previous interview studies of HTA agencies, such as to collaborate with other organizations and to be attentive to implementation considerations. Our interview study also provides a rich description of organizations supporting the use of research evidence, which can be drawn upon by those establishing or leading similar organizations in LMICs.

## Background

Organizations have been established in many countries and internationally to support the use of research evidence in developing health policy. These include organizations that produce clinical practice guidelines (CPGs), health technology assessment (HTA) agencies, and organizations that directly support the use of research evidence in developing health policy on an international, national, and state or provincial level (*i.e.*, government support units, or GSUs). As we argued in the introductory article in the series, a review of the experiences of such organizations, especially those based in low- and middle-income countries (LMICs) and that are in some way successful or innovative, can reduce the need to 'reinvent the wheel' and inform decisions about how best to organize support for evidence-informed health policy development processes in LMICs (Table 1) [1]. We described in the second article in the series the methods and findings from the first (survey) phase of our three-phase, multi-method study [2,3].

**Table 1:** Overview of the four-article series

[1]	Synthesis of findings from the three-phase, multi-method study
[2]	Survey of a senior staff member (the director or his or her nominee) of clinical practice guideline-producing organizations, HTA agencies, and government support units

**This article**	**Interview with the senior staff member of a purposively sampled sub-group of these three types of organizations, with an emphasis on those organizations that were particularly successful or innovative**

[21]	Case descriptions (based on site visits) of one or more organizations supporting the use of research evidence from among the cases described in the interviews and (once) other cases with which we were familiar, again with an emphasis on those organizations that were particularly successful or innovative

We focus here on describing the methods and findings from the study's second phase. In this phase, we interviewed the senior staff member of a purposively sampled sub-group of organizations around the world, and especially in LMICs, that are in some way successful or innovative in supporting the use of research evidence in the development of CPGs, HTAs, and health policy. Previous efforts to describe the experiences of such organizations have relied primarily on surveys [4-16], which offer the potential for capturing a tremendous breadth of experiences. Only a small number of efforts have relied on interviews and then only with HTA agencies (or in one case with individuals associated with both HTA and health-services research), not with CPG-producing organizations or GSUs [14,17-20]. Interviews offer the potential for capturing experiences in far more depth, particularly the experiences of organizations that may be under-represented in surveys, such as GSUs, organizations that are in some way successful or innovative, and organizations that are based in LMICs. In the next and final article in the series, we provide more detail about the methods and findings from the third (case descriptions) phase of the study [21].

## Methods

### Study sample

We purposively sampled organizations from among those who completed a questionnaire based on the following three criteria: 1) able to provide rich descriptions of their processes and lessons learned; 2) particularly successful or innovative in one or more of the seven domains covered in the questionnaire; and 3) influential over time within their own jurisdiction in supporting the use of research evidence, or influential in the establishment or evolution of similar organizations in other jurisdictions. The first criterion was applied by one member of the study team (RM) based on his reading of the completed questionnaires. The second and third criteria were applied by three members of the study team (AO, JNL, RM) based on their knowledge of and experience with these types of organizations.

### Interview guide development and interviewing

We developed the first draft of the semi-structured interview guide in parallel with the questionnaire as a mechanism to augment questions that could not or could only partially be addressed in the questionnaire. These 18 core questions were followed by organization-specific questions that arose based on responses provided in the questionnaire and by cross-cutting questions that addressed particular themes or hypotheses that emerged from the survey or earlier interviews. One member of the study team (RM) piloted the interview guide with four organizations, at least one of which was from each of the three categories. No significant changes were made after piloting. See 'Additional file 1: Interview guide' for the interview guide for units participating in the telephone interviews. A request to be interviewed was sent by email to the director (or another appropriate person) of each eligible organization and a date and time was set either through e-mail or telephone calls. The same member of the study team (RM) either conducted the interviews or supervised a trained interviewer who conducted the interviews (LJ). All interviews were conducted by telephone. Notes of all interviews were taken simultaneously. All interviews were audio-taped but only select interview segments were transcribed verbatim.

### Data management and analysis

Detailed summaries of each interview were prepared by one member of the study team (RM) using both the audio tapes and notes taken during the interviews, and these detailed summaries were subsequently analyzed independently by two members of the study team (AO, JL). The detailed summaries were organized by question. During the analysis, the detailed summaries were first read separately and supplemented, where necessary, by listening to part or all of the corresponding audio tapes. Binary or categorical responses to more structured questions were counted when possible. Themes were identified from among responses to semi-structured questions using a constant comparative method of analysis. Then question- and theme-specific groupings of the detailed summaries were developed and read, and the themes were modified or amplified. Illustrative quotations were identified to supplement the narrative description of the themes.

The principal investigator for the overall project (AO), who is based in Norway, confirmed that, in accordance with the country's Act on ethics and integrity in research, this study did not require ethics approval from one of the country's four regional committees for medical and health research ethics. We obtained verbal consent to participate in an interview. The nature of our request to participate in an interview made clear that we would be profiling particular organizations. We did not in any way indicate that we would treat interview data as confidential or that we would safeguard participants' anonymity. Nevertheless, we take care to ensure that no comments can be attributed to a single individual even if the organization about which an individual is speaking has been identified. We shared a report on our findings with participants and none of them requested any changes to how we present the data.

## Results

The director (or his or her nominee) was interviewed in 25 organizations, including five organizations that produce CPGs, three that produce HTAs, five that produce both CPGs and HTA, and 12 GSUs. Six organizations were in Western Europe, five in North America, four in Asia, three in Latin America, two each in Africa, Eastern Europe, and the Middle East, and one in Australia. The organizations varied in size from a few people to 50. No organizations declined to participate in the interviews.

See additional file 2: Qualitative data for additional qualitative data, particularly illustrative quotations, from the interviews. In the additional file, the text excised from this article is bolded. In this article, the location from which text was excised is denoted by a reference to the additional file.

### Mix of internally produced and externally commissioned work

The organizations employed a mix of models for producing outputs, with some undertaking some or all of the work internally and others commissioning some or all of the work externally. Seven organizations that produce CPGs, HTAs, or both commissioned little or no work (although one was soon to begin), five commissioned some work (up to 25%), and one commissioned most of its work. Six GSUs commissioned little or no work, four commissioned some work, and the other two commissioned about half their work.

### Focus of activities

There was substantial variation in the number and type of activities in which the organizations were involved. All but one of the CPG-producing organizations were involved only in producing CPGs, and the remaining organization was involved in the education of both physicians and consumers (patients and general public) as well. Most (5 of 7) of the organizations that produce HTAs, or both CPGs and HTAs, reported producing systematic reviews as their major activity, while three reported undertaking economic analyses and dissemination activities as well. Other activities undertaken by organizations that produce HTAs, or both CPGs and HTAs, included horizon scanning, preparing policy papers, and conducting evaluations (one each). GSUs reported involvement in a variety of activities, including producing systematic reviews (n = 3), conducting policy analyses (n = 3), training and capacity building (n = 3), producing CPGs (n = 2), conducting evaluations (n = 2), conducting economic analyses (n = 2), conducting health systems research (n = 2), and undertaking consultations and communication activities (n = 2).

### Priority-setting

All but one of the organizations producing CPGs, HTAs, or both used informal methods for setting priorities, whereas GSUs were more likely to respond to direct government requests. The exception among organizations producing CPGs, HTAs, or both used a scoring system, however, the organization's director added: 'Finally we ask: Is the technology compelling or not compelling? We find most decisions about prioritising are actually intuitive, so we have rolled this in. So, despite the scoring sheet, the most important decision-making about priorities for us is intuitive.' Among the organizations producing CPGs, HTAs, or both, one organization reported responding to government requests and four reported consulting with stakeholders. Other criteria that were considered include the frequency and severity of the problem, potential for improvement and cost of achieving the improvement, and avoiding duplication. About half of these organizations reported making decisions internally, and about the same proportion reported having a board or advisory group that sets priorities. Turning now to the GSUs, more than half of them (7 of 12) reported responding to requests for applications, two reported responding to perceived policy needs, and one reported making decisions through consultations involving staff and the Minister of Health. One had a board and one made five-year plans based on an external review.

### Methods used in producing a product or delivering a service

Organizations producing CPGs, HTAs, or both tended to conduct or use systematic reviews (12 of 13) and to have a manual that described the methods they use (11 of 13). Far fewer convened groups to develop CPGs or HTAs (5 of 13), took equity considerations into account (1 of 13), or had established a process for addressing conflicts of interest (1 of 13). Two organizations described primarily using secondary sources rather than conducting their own systematic reviews (see A.F.2). Only one of the five organizations that convened groups reported using a formal consensus method (the RAND method), and two of the other organizations described using some kind of interactive process with either clinicians or policymakers (see A.F.2). GSUs were less likely to conduct or use systematic reviews (3 of 12) and to have a manual that described the methods they use (4 of 12) and also more likely to report using non-systematic methods to review the literature (3 of 12). Several GSUs reported conducting economic analyses and using a variety of methods, including surveys, epidemiological studies, and qualitative studies. One GSU reported working with ethicists and addressing issues of equity (see A.F.2).

Using rigorous methods that are systematic and transparent (sometimes shortened to 'being evidence-based') was the most commonly cited strength among all organizations. Several organizations that produce CPGs, HTAs, or both referred specifically to using 'Cochrane methods,' one noted their use of a hierarchy of outcomes, and another noted their use of the Grading of Recommendations Assessment, Development and Evaluation (GRADE) system (see A.F.2). The weaknesses noted by most of these types of organizations were inadequate resources, more specifically insufficient numbers of skilled staff and time, together with using labour- and time-intensive processes that limit the number and quality of CPGs and HTAs that can be produced and updated. The GSUs, on the other hand, identified a range of different types of research or evaluation methods as additional strengths, including systematic reviews, measurement of health system performance, economic analyses, and surveys. Other strengths noted by GSUs included: having a small organization that can respond quickly, publishing drafts for public comment, maintaining close links with policymakers, and having independence and financial stability. The weaknesses that were identified by GSUs tended to be limitations of the methods used or how the methods were employed, including: not usually providing an exhaustive literature search or critical appraisal, 'just a systematic review often not being exactly what the audience wants,' use of casual 'vote counting' instead of a more rigorous approach to synthesizing research evidence, inaccuracies in long-term forecasting, and limitations in how health system performance is measured. GSUs also identified inadequate human resources and time as weaknesses.

### Recommendations or policy decisions related to their products

There was a great deal of variability both within and across CPG-producing organizations, HTA-producing organizations, and GSUs in who makes recommendations or policy decisions related to their products and the processes they use. For example, organizations producing CPGs, HTAs, or both in some jurisdictions have full responsibility for making policy decisions, whereas in other jurisdictions these decisions are made at the highest levels in the Ministry of Health. Two GSUs based outside of government acknowledged having little understanding of how policy decisions are made. Other GSUs based outside of government complained about the limited role of research evidence in policy decisions (see A.F.2). In contrast, none of the directors based in government spoke of the limited role of research evidence (see A.F.2). There was also variability in the perceived strengths and weaknesses of the processes that are used to make recommendations or policy decisions. Several directors referred to the explicit use of research evidence as a strength of the process and the time or capacity needed to produce recommendations as a weakness (see A.F.2). GSUs more consistently described their close links with policymakers as a strength, particularly those GSUs based in government, whereas organizations producing CPGs, HTAs, or both had conflicting viewpoints about such close links (see A.F.2). Two directors from organizations producing CPGs, HTAs, or both referred to the split between synthesizing the evidence and making a decision as a strength, whereas another director identified the involvement of stakeholders as a strength (see A.F.2). Another organization identified involvement of stakeholders as a weakness as well as a strength (see A.F.2). Two organizations producing CPGs, HTAs, or both noted their lack of influence as a weakness (see A.F.2). A lack of understanding of evidence-informed decision-making and the need for more education of and communication with policymakers was also noted (see A.F.2) Organizations sometimes mentioned the media as both a strength and a weakness in how recommendations or policy decisions related to their products are made (see A.F.2).

### Implementing recommendations or policy decisions related to their products

Most organizations argued that it is the clients who requested a CPG or HTA, the minister of health or more generally the department of health who is responsible for implementing recommendations or policy decisions related to their products (see A.F.2). Nearly all GSUs viewed policy implementation as the government's responsibility, although a couple of directors suggested that individual physicians also have some responsibility. Some organizations noted that responsibility for implementation is frequently spread among several organizations or that it is not clear who is responsible for implementing policy decisions. All types of organizations tended to focus largely on weaknesses in implementation, with few exceptions (see A.F.2). One reason that was frequently cited for this shortfall was the existence of multiple actors and multiple decision-makers in implementation processes that can be quite decentralized (see A.F.2). Other reasons that were cited for inadequate implementation included the general lack of formal processes for implementation, the specific challenges associated with guideline implementation (*e.g.*, lack of financial incentives for guideline adherence, practical difficulties in engaging health professionals, particularly those in rural areas), and the lack of funds to pay for effective (but expensive) technologies (see A.F.2).

### Approaches to personal communication with decision-makers

While informal relationships with policymakers were identified more frequently as important by GSUs (8 of 12) than by organizations producing CPGs, HTAs, or both (4 of 13), nearly all of the organizations reported using personal communications with decision-makers, particularly policymakers. For organizations producing CPGs, HTAs, or both, informal relationships with health professionals (8 of 13) and academics (5 of 13) were identified more frequently as important to their organization than relationships with policymakers, and informal relationships with other HTA organizations (*e.g.*, Agency for Healthcare Research and Quality, National Institute for Health and Clinical Excellence, and Scottish Intercollegiate Guidelines Network) (n = 3), the Cochrane Collaboration (n = 2), International Network of Agencies for Health Technology Assessment (n = 1), opinion leaders (n = 1), the health services (n = 1), and the public (n = 1) were identified less frequently as important to their organization. For GSUs, informal relationships with academics (n = 6) and health professionals (n = 3) were identified less frequently as important to their organization than relationships with policymakers, and informal relationships with advocacy organizations, non governmental organisations (NGOs), funders, industry, an HTA organization, and World Health Organisation (WHO) (one each) were identified even less frequently as important to their organization. Two organizations reported only having formal organizational relationships, and occasionally personal relationships, but no informal organizational relationships. While nearly all of the organizations reported using personal communications with decision-makers, a few organizations reported having only *ad hoc *communication, communication through policy advisors only, or only informal or indirect communication. A few of the organizations considered themselves to be decision-makers, and several others were located within government. Many of the organizations based within government viewed their close links with policymakers as a strength (see A.F.2). Organizations based outside of government also viewed their close relationships with policymakers as a strength (see A.F.2).

### Advocates and critics

Many organizations, particularly those producing CPGs, HTAs, or both, indicated that their strongest advocates were health professionals, including frontline clinicians and, especially, those who were involved in the organizations' activities (see A.F.2). However, physicians, particularly older physicians, specialists, and experts could also be among the most vocal critics (see A.F.2). The department of health, as well as other regulatory bodies, health insurers, and local health authorities or managers were also frequently identified as strong advocates, both by people working inside government and by those working in organizations based outside of government (see A.F.2). Other strong advocates that were identified included satisfied clients, the mass media, speciality societies, and other researchers. The last three were also seen as critics in some jurisdictions or in some circumstances. The most commonly identified critics were drug companies, particularly when their products were not recommended, and more generally 'groups who don't like our findings; for example, manufacturers or pharmaceuticals' (see A.F.2). Both other stakeholders and competitors were also frequently cited as critics. Stakeholders were generally perceived as critics when a new technology was not recommended (see A.F.2). Several organizations also identified as critics those who thought the processes took too long and cost too much and those with different methodological viewpoints (see A.F.2).

### Examples of successes and failures

Most of the examples of success among organizations producing CPGs, HTAs, or both were occasions where there was a perception that clinicians adhered to the organization's recommendations or policymakers based their decisions (at least in part) on the work of the organization. Only one organization producing CPGs, HTAs, or both could not identify an example of success, but on the other hand only one organization cited data from an audit to support the perception that clinicians adhered to the organization's recommendations. In three of the examples of policymakers acting on the work of an organization, an intervention was recommended, and policymakers' subsequent support for the intervention was perceived as a success (see A.F.2). In another three of the examples of policymakers acting on the work of an organization, an intervention was not recommended and policymakers' subsequent lack of support for the intervention was perceived as a success. One director cited a Minister's decision not to start a screening program, and a second cited a Minister's decision not to fund an expensive new technology, despite lobbying. A third director cited the example of a decision not to fund a drug and argued that this decision had saved lives and money (see A.F.2). Two examples of success were drawn from the field of public health: one that addressed smoking cessation, where success was attributed to good timing; the other addressed lowering the legal blood alcohol level for drivers.

The examples of success among GSUs were more diverse and the pathway from research evidence to policy more complex. Several organizations did not identify any examples of success or failure, noting that their role is only to report the research evidence, and the decision about whether and how to act on the research evidence is best left to others. The examples of success again tended to represent occasions where policymakers based their decisions (at least in part) on the work of the organization. One director cited examples of savings and improved accessibility to effective drugs from using generic drugs and supporting local producers. Another director cited savings from the discounts that could be negotiated based on drug class reviews. Other domains where success had been achieved included evaluations of a national health reform, healthcare financing policies, implementation of a human resources policy leading to re-categorising health professionals, provision of funds by a donor agency to support local coordination of HIV programs, and a housing policy.

The so-called failures typically involved the perception that clinicians were not adhering to the organization's recommendations, or policymakers were not basing their decisions (at least in part) on the work of the organization. Reasons ranged from insufficient awareness-raising among decision-makers to political lobbying by the patient groups, specialists, and companies directly affected by the decision. Often, the failures involved a technology not being recommended, but policymakers deciding to fund it anyway. However, one failure involved a technology being recommended but not being funded by government. Among the four examples of failures that pertained to broader health system policies, two recommendations were complex and a clear explanation was not offered as to why they were not acted upon (even though one would have saved the government money), one recommendation was likely not acted on because it was too broad, and one (involving cuts to the number of hospitals or to the number of beds within hospitals) was likely not acted on due to political opposition. Several other 'problems' were noted as well, such as insufficient research evidence, use of an intervention beyond its recommended uses, and inadequate monitoring of adherence to guidelines through audit (see A.F.2).

### Other strengths and weakness

When asked about any other strengths and weaknesses in how the organizations are organized, directors repeated many of the same strengths that were described previously (*e.g.*, independence, particularly from the pharmaceutical industry, close links to decision-makers, well trained and committed staff, use of rigorous methods, an interdisciplinary, collaborative approach, stakeholder involvement, and international collaboration), as well as many of the same weaknesses (*e.g.*, a lack of well trained staff, insufficient resources, inadequate international collaboration, the amount of time, energy, and resources required, and unrealistic expectations of clients). The relatively small size of the organizations was viewed by many organizations either as a strength or as both a strength and a weakness (see A.F.2). The relatively small size of the organizations and the relatively low pay of those working in the organizations were viewed by some organizations as a weakness (see A.F.2).

### Advice to others

The advice offered to those trying to establish similar organizations can be grouped into seven main recommendations.

#### Collaborate with other organizations

Most directors emphasised collaboration as important both in establishing an organization and in the ongoing work of an organization (see A.F.2).

#### Establish strong links with policymakers and involve stakeholders in the work

Many directors, particularly those working in GSUs, strongly recommended that organizations 'establish links to policymakers' (see A.F.2). A number of directors from across all types of organizations also stressed the importance of involving stakeholders (see A.F.2).

#### Be independent and manage conflicts of interest among those involved in the work

While many directors argued for establishing strong links with policymakers and involving stakeholders in the organization's work, a number of them highlighted the importance of being independent and managing conflicts of interest (see A.F.2).

#### Build capacity among those working in the organization

Many directors emphasised the challenge and the importance of recruiting or training multidisciplinary staff (see A.F.2). A couple of directors noted the importance of having a multidisciplinary team and, specifically in LMICs, thinking internationally (see A.F.2). Several directors, particularly those working in GSUs, emphasised the importance of leadership capacity (see A.F.2).

#### Use good methods and be transparent in the work

Many directors stressed the importance of using good methods and being transparent (see A.F.2).

#### Start small, have a clear audience and scope, and address important questions

A number of directors stressed the magnitude of the work involved, and hence the importance of starting small, having a clear audience and scope, and addressing important questions (see A.F.2). And while several directors pointed out the need to address important questions, no consistent advice emerged about how to approach the selection of questions (see A.F.2).

#### Be attentive to implementation considerations even if implementation is not a remit

Several directors noted the importance of implementation (see A.F.2). A number of directors who did not comment on implementation had made clear that implementation is not part of their organizations' work; however, some of these directors indicated that implementation considerations still inform their work even if responsibility for implementation lies elsewhere.

### Roles for WHO

Only a small number of directors provided comments about WHO's potential role. However, these comments almost always pertained to the role that WHO is or could be playing in fostering collaborations across organizations (see A.F.2).

## Discussion

### Principal findings from the interviews

The organizations employed a mix of models for producing outputs – with some undertaking some or all of the work internally and others commissioning some or all of the work externally – and there was substantial variation in the number and type of activities in which the organizations were involved. All but one of the organizations producing CPGs, HTAs, or both used informal methods for setting priorities, whereas GSUs were more likely to respond directly to government requests. Organizations producing CPGs, HTAs, or both were much more likely than GSUs to conduct or use systematic reviews and to have a manual that described the methods they use. Using rigorous methods that are systematic and transparent (sometimes shortened to 'being evidence-based') was the most commonly cited strength among all organizations, whereas organizations producing CPGs, HTAs, or both noted inadequate resources coupled with using labour- and time-intensive processes as weaknesses, and GSUs noted limitations of the methods used or how the methods were employed as weaknesses.

There was a great deal of variability in who makes recommendations or policy decisions related to the organizations' products, the processes they use, and the perceived strengths and weaknesses in these processes. Several organizations referred to the explicit use of research evidence as a strength of the processes, and the time or capacity needed to produce recommendations as a weakness. GSUs more consistently described their close links with policymakers as a strength, particularly those GSUs based in government, whereas organizations producing CPGs, HTAs, or both had conflicting viewpoints about such close links. Most organizations argued that it is the clients who requested a CPG or HTA, the minister of health or more generally the department of health who is responsible for implementing recommendations or policy decisions related to their products. With few exceptions, all types of organizations tended to focus largely on weaknesses in implementation, rather than strengths. While informal relationships with policymakers were identified more frequently as important by GSUs than by organizations producing CPGs, HTAs, or both, nearly all of the organizations reported using personal communications with decision-makers, particularly policymakers, and many of the organizations viewed their close links with policymakers as a strength. While health professionals (particularly those involved in the organizations' activities) and policymakers were often identified as advocates, and drug companies, patient groups, and competitors were often identified as critics, particular sub-groups could be supportive or critical depending on their perception of the organizations' general focus (*e.g.*, threatening professional freedom, diminishing the role of expertise, creating funding pressures, and enhancing accountability), its specific approach (*e.g.*, including some forms of research evidence but not others, consulting broadly with affected groups, taking too long or charging a fee to produce a report, and producing reports that are difficult to understand), and its specific recommendations on any given topic (*e.g.*, recommending against providing, covering or reimbursing a technology).

Most of the examples of success among organizations producing CPGs, HTAs, or both were occasions where there was a perception that clinicians adhered to the organization's recommendations, or policymakers based their decisions (at least in part) on the work of the organization. The examples of so-called success among GSUs were more diverse, and the pathway from research evidence to policy more complex. The so-called failures typically involved the perception that clinicians were not adhering to the organization's recommendations, or policymakers were not basing their decisions (at least in part) on the work of the organization. Reasons ranged from insufficient awareness-raising among decision-makers to political lobbying by the patient groups, specialists, and companies directly affected by the decision. The advice offered to those trying to establish similar organizations can be grouped into seven main recommendations: 1) collaborate with other organizations; 2) establish strong links with policymakers and involve stakeholders in the work; 3) be independent and manage conflicts of interest among those involved in the work; 4) build capacity among those working in the organization; 5) use good methods and be transparent in the work; 6) start small, have a clear audience and scope, and address important questions; and 7) be attentive to implementation considerations even if implementation is not a remit. Only a small number of directors provided comments about WHO's potential role, however, these comments almost always pertained to the role that WHO (or another international organization or network) is or could be playing in fostering collaborations across organizations.

### Strengths and weaknesses of the interviews

The interviews have three main strengths: 1) we drew on a regionally diverse project reference group to ensure that our draft protocol and interview guide were fit for purpose; 2) we interviewed roughly equal numbers of CPG- and HTA-producing organizations and GSUs; and 3) no organization declined to participate in the interviews. The interviews have three main weaknesses: 1) despite significant efforts to identify organizations in low- and middle-income countries, just under one-half (48%) of the organizations we interviewed were drawn from high-income countries; 2) despite efforts to ask questions in neutral ways, many organizations may have been motivated by a desire to tell us what they thought we wanted to hear (*i.e.*, there may be a social desirability bias in their responses); and 3) given the nature of many of the structured questions posed and responses given the analysis relied heavily on counting and hence could have missed subtleties in emphasis and inadvertent omissions of select points.

### What the interviews add

The findings from our interview study, the most broadly based of its kind, extend the applicability of the messages arising from previous interview studies of HTA agencies to both CPG-producing organizations and GSUs. First, our findings concur with several conclusions from an interview study focused on prominent individuals associated with HTA and health services research in Canada in 1999 [17]. The study found that: 'A key question now being asked by policymakers – implicitly if not explicitly – concerns the value for money from funding HTA organizations. Might funds not be spent better on other activities?' Interviewees acknowledged insufficiencies in their ability to document their value relative to their budgets. The conclusions that were or can be drawn from this finding support the advice to collaborate with other organizations and, indirectly, the advice to establish strong links with policymakers, involve stakeholders in the work, and to be attentive to implementation considerations even if implementation is not a remit. Second, our findings concur with two conclusions that were or can be drawn from an interview study (which, like ours, followed a survey) focused on European HTA agencies that participated in a collaborative project called EUR-ASSESS [14]. The study found a wide diversity of approaches and highlighted the importance of collaboration and shared learning, which is consistent with the advice to collaborate with other organizations. The study also provided support for the advice to be attentive to implementation considerations. Third, our findings concur with four conclusions that were or can be drawn from an interview study focused on the directors and staff from six Canadian HTA agencies [18,20]. In terms of the production of HTAs, the study found tensions between: 1) standardising and streamlining production and diversifying outputs; 2) contextualising results and sharing work across agencies; 3) addressing a large scope and addressing one well-delineated question; and 4) doing more and producing less measurable outputs [18]. In terms of the dissemination of HTAs, the study found that although the HTA agencies had recognised that dissemination activities need to be intensified, why and how particular approaches should be adopted was still under debate [20]. A parallel interview study of HTA users found that significant organizational, scientific, and material limitations hinder the use of scientific evidence and suggested that overcoming such barriers requires a greater commitment from both HTA producers and users [19]. The conclusions that were or can be drawn from these three related studies are consistent with the advice to collaborate with other organizations, to involve stakeholders in the work, to use good methods and be transparent in the work, and to be attentive to implementation considerations. Our interview study also provides a rich description of the structure, processes, outputs, and perceived strengths and weaknesses of CPG-producers and GSUs as well as HTA-producers, which can be drawn upon by those establishing or leading similar organizations in LMICs.

### Implications for policymakers and for international organizations and networks

As we argued in the first article in the series, policymakers can play a strong supporting role for these organizations, both by building strong links with the organizations while respecting their independence and by encouraging them to follow the recommendations that emerged from the study, such as to collaborate with other organizations. WHO and international networks such as the Guidelines International Network and the International Network of Agencies for Health Technology Assessment have an important role to play in fostering collaboration among organizations, particularly collaboration that brings together well-established organizations with organizations that are new or have only limited capacity. The reliance of many organisations (particularly those producing CPGs and HTAs) on systematic reviews suggests that other international networks, such as the Cochrane Collaboration, have important roles to play both in conducting and keeping up-to-date systematic reviews that address important high-priority policy questions and in building capacity to undertake systematic reviews that address such questions.

### Implications for future research

Given that timing or timeliness emerged as one of two factors that increase the prospects for research use in policymaking, and that the labour- and time-intensiveness of the processes used was a commonly cited weakness, research is needed to develop methods and organizational structures to respond rapidly to policymakers' questions [22]. There is also a need for research about balancing the need for strong links with policymakers on the one hand and the need for independence and managing conflicts of interest on the other, and for research about supporting the use of reports and the implementation of their recommendations.

## Competing interests

The authors declare that they have no financial competing interests. The study reported herein, which is the second phase of a larger three-phase study, is in turn part of a broader suite of projects undertaken to support the work of the World Health Organization (WHO) Advisory Committee on Health Research (ACHR). Both JL and AO are members of the ACHR. JL is also President of the ACHR for the Pan American Health Organization (WHO's regional office for the Americas). The Chair of the WHO ACHR, a member of the PAHO ACHR, and several WHO staff members were members of the project reference group and, as such, played an advisory role in study design. Two of these individuals provided feedback on the penultimate draft of the report on which the article is based. The authors had complete independence, however, in all final decisions about study design, in data collection, analysis and interpretation, in writing and revising the article, and in the decision to submit the manuscript for publication.

## Authors' contributions

JL participated in the design of the study, participated in analyzing the qualitative data, and drafted the article and the report in which it is based. AO conceived of the study, led its design and coordination, participated in analyzing the qualitative data, and contributed to drafting the article. RM participated in the design of the study, led the data collection and the analysis of the qualitative data, and contributed to drafting the article. EP contributed to data collection. All authors read and approved the final manuscript.

## Supplementary Material

Additional file 1 **Interview guide for units participating in the telephone interviews.** Click here for file

Additional file 2 **Qualitative data from the interviews.** Click here for file

## References

[B1] Lavis JN, Oxman AD, Moynihan R, Paulsen EJ (2008). Evidence-informed health policy 1 – Synthesis of findings from a multi-method study of organizations that support the use of research evidence. Implementation Science.

[B2] Lavis JN, Paulsen EJ, Oxman AD, Moynihan R (2008). Evidence-informed health policy 2 – Survey of organizations that support the use of research evidence. Implementation Science.

[B3] Moynihan R, Oxman AD, Lavis JN, Paulsen E (2008). Evidence-Informed Health Policy: Using Research to Make Health Systems Healthier – Report from the Kunnskapssenteret (Norwegian Knowledge Centre for the Health Services), No 1-2008.

[B4] Audet A, Greenfield S, Field M (1990). Medical practice guidelines: Current activities and future directions. Annals of Internal Medicine.

[B5] McGlynn EA, Kosecoff J, Brook RH (1990). Format and conduct of consensus development conferences. International Journal of Technology Assessment in Health Care.

[B6] Grol R, Eccles M, Maisonneuve H, Woolf S (1998). Developing clinical practice guidelines: The European experience. Disease Management and Health Outcomes.

[B7] Engelbrecht R, Courte-Wienecke S (1999). A Survey on the Current State of Development, Dissemination and Implementation of Guidelines of Clinical Practice in European countries.

[B8] Woolf SH, Grol RP, Hutchinson A, Eccles M, Grimshaw JM (1999). Potential benefits, limitations, and harms of clinical guidelines. British Medical Journal.

[B9] The Appraisal of Guidelines, Research and Evaluation in Europe (AGREE) Collaborative Group (2000). Guideline development in Europe: An international comparison. International Journal of Technology Assessment in Health Care.

[B10] Burgers JS, Grol R, Klazinga NS, Makela M, Zaat J, AGREE Collaboration (2003). Towards evidence-based clinical practice: An international survey of 18 clinical guideline programs. International Journal for Quality in Health Care.

[B11] Graham ID, Beardall S, Carter AO, Tetroe J, Davies B (2003). The state of the science and art of practice guidelines development, dissemination and evaluation in Canada. Journal of Evaluation in Clinical Practice.

[B12] Perry S, Gardner E, Thamer M (1997). The status of health technology assessment worldwide: Results of an international survey. International Journal of Technology Assessment in Health Care.

[B13] Perry S, Thamer M (1997). Health technology assessment: Decentralized and fragmented in the US compared to other countries. Health Policy.

[B14] Sassi F (2000). The European way to health technology assessment. Lessons from an evaluation of EUR-ASSESS. International Journal of Technology Assessment in Health Care.

[B15] Hastings J, Adams EJ (2006). Joint project of the international network of agencies for health technology assessment – Part 1: Survey results on diffusion, assessment, and clinical use of positron emission tomography. International Journal of Technology Assessment in Health Care.

[B16] Lavis JN, Robertson D, Woodside JM, McLeod CB, Abelson J (2003). How can research organizations more effectively transfer research knowledge to decision makers?. Milbank Quarterly.

[B17] McDaid D (2003). Co-ordinating health technology assessment in Canada: A European perspective. Health Policy.

[B18] Lehoux P, Tailliez S, Denis J-L, Hivon M (2004). Redefining health technology assessment in Canada: Diversification of producers and contextualization of findings. International Journal of Technology Assessment in Health Care.

[B19] Hivon M, Lehoux P, Denis J-L, Tailliez S (2005). Use of health technology assessment in decision making: Coresponsibility of users and producers?. International Journal of Technology Assessment in Health Care.

[B20] Lehoux P, Denis J-L, Tailliez S, Hivon M (2005). Dissemination of health technology assessment: Identifying the visions guiding an evolving policy innovation in Canada. Journal of Health Politics, Policy and Law.

[B21] Lavis JN, Moynihan R, Oxman AD, Paulsen EJ (2008). Evidence-informed health policy 4 – Case descriptions of organizations that support the use of research evidence. Implementation Science.

[B22] Lavis JN, Davies HTO, Oxman AD, Denis J-L, Golden-Biddle K, Ferlie E (2005). Towards systematic reviews that inform health care management and policy-making. Journal of Health Services Research and Policy.

