# Supplementary material for: Evidence-informed health policy 3 – Interviews with the directors of organizations that support the use of research evidence
Source: Implement Sci. 2008 Dec 17;3:55. doi: 10.1186/1748-5908-3-55 (PMC2639615; doi:10.1186/1748-5908-3-55)
Supplement: Additional file 1 — Interview guide for units participating in the telephone interviews. [file 1748-5908-3-55-S1.doc]

ID Number

Name of Interviewee

Position

Name of Unit

Description of Unit

1. What are the most important informal relationships the unit has that help make its work more effective? (*e.g.*, relationships that operate nationally and internationally, with government, with academic institutions, professional groups, etc.)
2. What kind of staff does your unit have and how does this impact on the work you do?
3. To what extent does the unit commission work internally, externally?
4. What is the range of activities the unit is involved in, and what proportion of its resources goes to each: *i.e.*, systematic reviews, HTAs, economic analysis, CPGs, preparing policy briefs and background documents for government, implementation and evaluation?
5. How are the priorities of the unit decided?
6. Can you describe in detail the methods your unit uses to produce the materials it produces? (*i.e.,* steps to developing a systematic review or HTA of CPG or policy brief)
7. Does the unit have a manual describing the methods its uses?
8. Can you talk more about the strengths and weaknesses of the methods your unit uses?
9. To what extent does your unit use personal communications with decision-makers?
10. Who makes policy decisions or recommendations in connection with the outputs of your unit and what process do they use?
11. What are the main strengths and weaknesses of the way policy decisions or recommendations are made?
12. Who is responsible for implementing policy decisions or recommendations?
13. What are the main strengths and weaknesses of that implementation?
14. Can you tell us more about the strongest advocates for your unit, and what they say, and about the strongest critics of the unit and what they say? (Get contacts if haven’t already.)
15. Can you give an example of a successful recommendation or decision that flowed from the work of your unit, and what made it successful?
16. Conversely, can you give an example of an unsuccessful recommendation or decision that flowed from the work of your unit, and what made it unsuccessful?
17. Can you talk more about advice you would give others trying to establish similar units? (Both positive advice and what pitfalls to avoid?)
18. Can you talk more about the strengths and weaknesses of how your unit is organised?
19. Follow up-questions - if needed - clarifying survey responses and asking any questions that may arise during interview.
