# Supplementary material for: Evidence-informed health policy 3 – Interviews with the directors of organizations that support the use of research evidence
Source: Implement Sci. 2008 Dec 17;3:55. doi: 10.1186/1748-5908-3-55 (PMC2639615; doi:10.1186/1748-5908-3-55)
Supplement: Additional file 2 — Qualitative data from the interviews. [file 1748-5908-3-55-S2.doc]

**Evidence-informed health policy: 3. Interviews with the directors of organizations that support the use of research evidence. [Excerpt – Qualitative data from the interviews with text excised from the main text bolded]**

John N. Lavis^1^*, Andrew D. Oxman^2^, Ray Moynihan^3^, Elizabeth J. Paulsen^2^

*Corresponding author

^1^Centre for Health Economics and Policy Analysis, Department of Clinical Epidemiology and Biostatistics, and Department of Political Science, McMaster University, 1200 Main St. West, HSC-2D3, Hamilton, ON, Canada L8N 3Z5

^2^Norwegian Knowledge Centre for the Health Services, Pb. 7004, St. Olavs plass, Oslo, Norway N-0130

^3^School of Medicine and Public Health, Faculty of Health, The University of Newcastle, Medical Sciences Building – Level 6, Callaghan, NSW, Australia 2308

Email addresses:

JNL: lavisj@mcmaster.ca

ADO: oxman@online.no

RM: ray.moynihan@newcastle.edu.au

EJP: elizabeth.paulsen@kunnskapssenteret.no

**Mix of internally produced and externally commissioned work**

The organizations employed a mix of models for producing outputs, with some undertaking some or all of the work internally, and others commissioning some or all of the work externally. Seven organizations that produce CPGs, HTAs, or both commissioned little or no work (although one was soon to begin), five commissioned some work (up to 25%), and one commissioned most of its work. Six GSUs commissioned little or no work, four commissioned some work, and the other two commissioned about half their work.

#### Focus of activities

There was substantial variation in the number and type of activities in which the organizations were involved. All but one of the CPG-producing organizations were involved only in producing CPGs, and the remaining organization was involved in the education of both physicians and consumers (patients and general public) as well. Most (5 of 7) of the organizations that produce HTAs or both CPGs and HTAs reported producing systematic reviews as their major activity, while three reported undertaking economic analyses and dissemination activities as well. Other activities undertaken by organizations that produce HTAs or both CPGs and HTAs included horizon scanning, preparing policy papers, and conducting evaluations (one each). GSUs reported involvement in a variety of activities, including producing systematic reviews (n = 3), conducting policy analyses (n = 3), training and capacity building (n = 3), producing CPGs (n = 2), conducting evaluations (n = 2), conducting economic analyses (n = 2), conducting health systems research (n = 2), and undertaking consultations and communication activities (n = 2).

#### Priority-setting

All but one of the organizations producing CPGs, HTAs, or both used informal methods for setting priorities, whereas GSUs were more likely to respond to direct government requests. The exception among organizations producing CPGs, HTAs, or both used a scoring system, however, the organization’s director added: ‘Finally we ask: Is the technology compelling or not compelling? We find most decisions about prioritising are actually intuitive, so we have rolled this in. So, despite the scoring sheet, the most important decision-making about priorities for us is intuitive.’ Among the organizations producing CPGs, HTAs, or both, one organization reported responding to government requests and four reported consulting with stakeholders. Other criteria that were considered include the frequency and severity of the problem, potential for improvement and cost of achieving the improvement, and avoiding duplication. About one-half of these organizations reported making decisions internally, and about the same proportion reported having a board or advisory group that sets priorities. Turning now to the GSUs, more than half of them (7 of 12) reported responding to requests for applications, two reported responding to perceived policy needs, and one reported making decisions through consultations involving staff and the Minister of Health. One had a board, and one made five-year plans based on an external review.

#### Methods used in producing a product or delivering a service

Organizations producing CPGs, HTAs, or both tended to conduct or use systematic reviews (12 of 13) and to have a manual that described the methods they use (11 of 13). Far fewer convened groups to develop CPGs or HTAs (5 of 13), took equity considerations into account (1 of 13), or had established a process for addressing conflicts of interest (1 of 13). Two organizations described primarily using secondary sources rather than conducting their own systematic reviews. **One director said: ‘We look to secondary sources and based on those we write the final guideline. We don’t have staff to do full searches. We think we are still producing good quality evidence-based recommendations, without having to go through 6000 papers.’ Another reported: ‘If we haven’t got good secondary source material, we go to primary, but that’s rare.’** Only one of the five organizations that convened groups reported using a formal consensus method (the RAND method), and two of the other organizations described using some kind of interactive process with either clinicians or policymakers. **The one organization that takes into account issues of equity said: ‘The INCLEN method introduces equity. That’s one of the aspects of recent guidelines that many of panel members were not able to grasp fully until the end of the process, but many of them were able to understand at the end of the process.’ The one organization that had a process for addressing conflicts of interest said:**

**‘The conflicts of interest, that’s a modification that we brought. We tried to address conflicts of interest at the very start, during organization. We are strict with the research committee, who are appraising evidence. We requested them to divest from conflicts of interest, and we actually graded conflicts of interest, especially from the pharmaceutical industry.’**

GSUs were less likely to conduct or use systematic reviews (3 of 12) and to have a manual that described the methods they use (4 of 12), and were more likely to report using non-systematic methods to review the literature (3 of 12). Several GSUs reported conducting economic analyses and using a variety of methods, including surveys, epidemiological studies, and qualitative studies. One GSU reported working with ethicists and addressing issues of equity. **Another GSU described using a highly interactive approach.**

**‘We clarify the research questions, assemble our own team, call them for a meeting, ask senior management what they want answered. Following that we do our literature search. . . We go back to our senior management and talk again. Then we design our research and bring in necessary competence as needed. We present our methodology to management and once cleared, we proceed with the research. We then do work and present it to different groups, including groups on ‘the ground’. We get as much support as we can from all the groups. . . We present our findings back to senior management as a draft.’**

Using rigorous methods that are systematic and transparent (sometimes shortened to ‘being evidence-based’) was the most commonly cited strength among all organizations. Several organizations that produce CPGs, HTAs, or both referred specifically to using ‘Cochrane methods,’ one noted their use of a hierarchy of outcomes, and another noted their use of the Grading of Recommendations Assessment, Development and Evaluation (GRADE) system. **The latter director said: ‘The good thing about GRADE is that it is an honest system which enables you to admit errors or weaknesses. The whole organization discusses and goes through what is going on every week, and questions things.’ The other strengths noted by these types of organizations included using secondary sources (two organizations), ‘pairing health professionals with our people who have good systematic review skills,’ independence from the pharmaceutical industry, and the ability to work with other groups in different countries to avoid duplication of efforts.** The weaknesses noted by most of these types of organizations were inadequate resources, more specifically insufficient numbers of skilled staff and time, together with using labour- and time-intensive processes that limit the number and quality of CPGs and HTAs that can be produced and updated.

The GSUs, on the other hand, identified a range of different types of research or evaluation methods as additional strengths, including systematic reviews, measurement of health system performance, economic analyses, and surveys. Other strengths noted by GSUs included: having a small organization that can respond quickly, publishing drafts for public comment, maintaining close links with policymakers, and having independence and financial stability. The weaknesses that were identified by GSUs tended to be limitations of the methods used or how the methods were employed, including: not usually providing an exhaustive literature search or critical appraisal, ‘just a systematic review often not being exactly what the audience wants,’ use of casual ‘vote counting’ instead of a more rigorous approach to synthesizing research evidence, inaccuracies in long-term forecasting, and limitations in how health system performance is measured. GSUs also identified inadequate human resources and time as weaknesses.

#### Recommendations or policy decisions related to their products

There was a great deal of variability both within and across CPG-producing organizations, HTA-producing organizations, and GSUs in who makes recommendations or policy decisions related to their products and the processes they use. For example, organizations producing CPGs, HTAs, or both in some jurisdictions have full responsibility for making policy decisions, whereas in other jurisdictions these decisions are made at the highest levels in the Ministry of Health. Two GSUs based outside of government acknowledged having little understanding of how policy decisions are made. Other GSUs based outside of government complained about the limited role of research evidence in policy decisions. **One organization said: ‘However, in any political environment, the outcomes are influenced by pressure and political groups, so the quality of the policy decision may be watered down.’ Another director said there is ‘no clear or ongoing structure for policy to be informed by our research.’** In contrast, none of the directors based in government spoke of the limited role of research evidence **and one said: ‘Despite evidence, we need to have political understanding, so we need a mixture of both.’**

There was also variability in the perceived strengths and weaknesses of the processes that are used to make recommendations or policy decisions. Several directors referred to the explicit use of research evidence as a strength of the process, and the time or capacity needed to produce recommendations as a weakness. **One person noted: ‘By time the unit produces something, the Minister has changed or the issue is no longer on the agenda or there is an election coming up, so there is a discrepancy between the “calendars” of the Minister and the unit.’ Another director, describing the lack of capacity said: ‘The difficult thing in this whole area is that it is early days for this type of reviewing technology. It’s like when we had the first car; it took a long time for the industry to take off. It’s still early days. I can’t hire people who are ready to do this work.’**

GSUs more consistently described their close links with policymakers as a strength, particularly those GSUs based in government, whereas organizations producing CPGs, HTAs, or both had conflicting viewpoints about such close links. **One director from a GSU based in government said: ‘A strength is that although there are no formal structures, there is a lot of personal contact.’ Another said: ‘Senior management have started to understand evidence-based information.’** Two directors from organizations producing CPGs, HTAs, or both referred to the split between synthesizing the evidence and making a decision as a strength, whereas another director identified the involvement of stakeholders as a strength**, and a fourth said: ‘One advantage is that we are very closely affiliated with the Ministry of Health. We have shared staff so our recommendations are accepted fairly automatically. We don’t have to go through a courting process. We are part of the process.’** Another organization identified involvement of stakeholders as a weakness as well as a strength: **‘The problem with groups is that sometimes they have other agendas, particularly with outside experts. . . Sometimes key experts are very eager to get new procedure out.’** Two organizations producing CPGs, HTAs, or both noted their lack of influence as a weakness. **One said: ‘One weakness is that the realm of influence is very small.’ The other noted that: ‘We as an organization play very little role in terms of influencing policy. We have an interest and mission in doing this, but are not successful at doing this. We are more successful at the clinical level.’**

A lack of understanding of evidence-informed decision-making and the need for more education of and communication with policymakers was also noted. **‘The evidence world is not really joined up yet. It’s really difficult to do EBM [evidence-based medicine] at all because not many people really understand how it works or the impact it can have on organizations. There is a lot of education involved and the policymakers themselves may not have a great understanding of evidence on what it can and can’t do.’ Another person linked the need for better understanding of evidence with the ability of vested interests to influence what happens: ‘Limitation of public funds keeps us from being totally able to communicate the nature of the use of evidence, so without that . . . it’s easy for vested interests to criticize.’**

Organizations sometimes mentioned the media as both a strength and a weakness in how recommendations or policy decisions related to their products are made. **One director described the attention given to its reports by the mass media as a strength, but the nature of their reporting as a weakness. ‘We need more professional reporting in the media, to understand the indicators. They can exploit much more what we are reporting. This is a weakness of the media.’ Another director added:**

**‘The media is a double-edged sword. We don't produce press releases, but quite often journalists may come to us for more information. You may produce a piece of research, which may have statistically significant findings. But you know with research you can easily lose control of the implications of your research, because we know that it can be overturned by another piece of research. Media coverage is good, but often media doesn’t understand the subtlety of what we are doing, and the application of the research can be misunderstood and misrepresented.’**

#### Implementing recommendations or policy decisions related to their products

Most organizations argued that it is the clients requesting a CPG or HTA, the minister of health or more generally the department of health who is responsible for implementing recommendations or policy decisions related to their products. **One director said: ‘The organization is not allowed to make recommendations, only provide information. Whoever asks for information, it is their responsibility to take it forward and do something with it.’ Another director said: ‘We have very little control of whether recommendations are implemented. It can be quite frustrating. That’s a major drawback. I can’t think of a project where we did implementation. I think this is a limitation. On other hand, to implement recommendations could require a lot of manpower and we don’t have the authority.’** Nearly all GSUs viewed policy implementation as the government’s responsibility, although a couple of directors suggested that individual physicians also have some responsibility. Some organizations noted that responsibility for implementation is frequently spread among several organizations or that it is not clear who is responsible for implementing policy decisions.

All types of organizations tended to focus largely on weaknesses in implementation, with few exceptions. **The following statement reflects the tenor of many responses: ‘Generally the dissemination of policy and link with policy implementation is not as strong as we would like to see.’** One reason that was frequently cited for this shortfall was the existence of multiple actors and multiple decision-makers in implementation processes that can be quite decentralized. **One director said: ‘a weakness appears to be that the implementation of policy decisions or recommendations is that it is pretty fractured. This is because the different health authorities or governing bodies take advice from different sources, so there appears to be variation in practice.’** Other reasons that were cited for inadequate implementation included the general lack of formal processes for implementation, the specific challenges associated with guideline implementation (*e.g.*, lack of financial incentives for guideline adherence, practical difficulties in engaging health professionals, particularly those in rural areas), and the lack of funds to pay for effective (but expensive) technologies.

**Some organizations saw both strengths and weaknesses in particular approaches to implementation. One director said: ‘A strength is that it starts at the top, with a direction to implement, across hospitals, for example,’ but added ‘a major weakness of this system is that it is asking someone who has no ownership of the guidelines to implement it.’ Another director noted that: ‘Where the Ministry has decided which technologies are going to be incorporated in the national list . . . this is regulated to make sure that this is funded for,’ but added: ‘Sometimes, there is dissension about why certain technologies aren’t added and they are expensive.’ A third director identified that: ‘The main strength is that a decision has been made by the Minister and backed by Cabinet. The actual implementation flows quickly and that’s good.’ But the director added: ‘The weaknesses have to do with the process being watered down by certain stakeholder interests. At this stage, there is still quite an imbalance in the way decisions are handled at national level and at the provincial level.’**

#### Approaches to personal communication with decision-makers

While informal relationships with policymakers were identified more frequently as important by GSUs (8 of 12) than by organizations producing CPGs, HTAs, or both (4 of 13), nearly all of the organizations reported using personal communications with decision-makers, particularly policymakers. For organizations producing CPGs, HTAs, or both, informal relationships with health professionals (8 of 13) and academics (5 of 13) were identified more frequently as important to their organization than relationships with policymakers, and informal relationships with other HTA organizations (*e.g.,* Agency for Healthcare Research and Quality, National Institute for Health and Clinical Excellence, and Scottish Intercollegiate Guidelines Network) (n = 3), the Cochrane Collaboration (n = 2), International Network of Agencies for Health Technology Assessment (n = 1), opinion leaders (n = 1), the health services (n = 1), and the public (n = 1) were identified less frequently as important to their organization. For GSUs, informal relationships with academics (n = 6) and health professionals (n = 3) were identified less frequently as important to their organization than relationships with policymakers, and informal relationships with advocacy organizations, non governmental organisations (NGOs), funders, industry, an HTA organization, and World Health Organisation (WHO) (one each) were identified even less frequently as important to their organization. Two organizations reported only having formal organizational relationships and occasionally personal relationships but no informal organizational relationships. While nearly all of the organizations reported using personal communications with decision-makers, a few organizations reported having only *ad hoc* communication, communication through policy advisors only, or only informal or indirect communication. A few of the organizations considered themselves to be decision-makers, and several others were located within government.

Many of the organizations based within government viewed their close links with policymakers as a strength. **‘This is a very strong area for us — the fact that we are embedded in government and report directly to the Deputy Minister is what helps. It creates a continuum between the recommendation and the policy decision at the end. We are often called upon by government to explain our work. We don’t seek to be policy decision-makers but we do influence things strongly because of our contacts with government. Sometimes, we are in touch with government on a daily or hourly basis.’ Another person working within government responded: ‘The importance the Minister gives to evaluation is not random. We are part of a government that came to power through a big democratisation process. We were lucky to get rid of a very authoritarian way. [This government places] big demands on accountability. One of most powerful instruments of accountability is evaluation.’**

Organizations based outside of government also viewed their close relationships with policymakers as a strength. **‘Every month we have a two hour meeting involving all decision-makers. We get them on the phone and make sure we are meeting their requirements. Also, we have some meetings to establish key priorities and so on.’ Another responded: ‘This is a very important component and we are basically constantly exchanging our ideas with respective departments at ministry and national levels, so our informal network is quite important for our research. Policymakers come to us for information and we feel we have access to these people through this contact.’ A third director said: ‘We have to, because of the question setting issues I mentioned. You need to understand what people need.’**

#### Advocates and critics

Many organizations, particularly those producing, indicated that their strongest advocates were health professionals, including front-line clinicians and, especially, those who were involved in the organizations’ activities. **One director said: ‘Our strongest advocates are healthcare professionals who have been on a guideline group and have had contact with us.’** However, physicians, particularly older physicians, specialists and experts, could also be among the most vocal critics. **One director said: ‘The strong critics are maybe the older physicians that do not want to start the process to develop evidence or guidelines. There is more opposition from this kind of physician.’ A second director said: ‘The strongest critics are the elderly people who first of all can’t apply evidence-based medicine, who call themselves experts.’ A third director identified a more general source of criticism: ‘Generally, negative responses come from health professionals who don’t see the guidelines as necessary and think that they get in the way of their clinical freedom.’**

The department of health, as well as other regulatory bodies, health insurers, and local health authorities or managers were also frequently identified as strong advocates, both by people working inside government and by those working in organizations based outside of government. **Policymakers could also be critical: ‘Critics would include the Minister and Director of Social Security, who say we take too much time and that our documents are difficult to translate into simple language.’ Another director noted mixed reactions from government: members of ‘the management board of government are cagey because they see the [work we do] as a pressure. On the other hand, the provincial auditors are very pleased because they feel it is an important source of accountability.’** Other strong advocates that were identified included satisfied clients, the mass media, speciality societies, and other researchers. The last three were also seen as critics in some jurisdictions or in some circumstances.

The most commonly identified critics were drug companies, particularly when their products were not recommended, and more generally ‘groups who don’t like our findings; for example, manufacturers or pharmaceuticals.’ **One director from a organization producing CPGs said: ‘We are best friends whenever we recommend a product. We are worst enemies when we say products should not be used. We are independent with no money from the pharmaceutical industry. They were interested in offering us a lot of money.’ Another person, commenting on both drug companies and specialists, said: ‘The strongest critics, often times it’s manufacturers, followed by specialists, well from manufacturers. We receive criticism because they are not happy when assessments are not in their favour. That’s very straightforward. With specialists it’s more subtle. The way it’s perceived, they know more about their area. They resent that anyone would interfere or try to assess.’**

Both other stakeholders and competitors were also frequently cited as critics. Stakeholders were generally perceived as critics when a new technology was not recommended**: ‘Some stakeholder groups are wary. They are happy when [we] say ‘yes’ to their new technology proposal based on the work we have done, but not if the answer is ‘no’.’ Similarly, another director said: ‘Naturally there will always be members of the public upset with decisions . . . because there is never enough money to put into everything, so the media and the public will always be critical of those left out.’ One person noted some competitors questioning whether the organization should produce their own guidelines when another organization was already producing guidelines with ten times the budget. Another director said: ‘Our major critics would be some of the organizations who have been around the same time but are more mainstreamed. Cochrane was critical at one stage. . .’ Another noted that ‘the critics can be from academia, strong competitors, they are less consulted, less involved.’ A fourth director said ‘the critics would say we have assumed a monopoly position in the region, which would make it difficult for neighbour countries in the region to get funding for interesting research projects.’**

Several organizations also identified as critics those who thought the processes took too long and cost too much and those with different methodological viewpoints. **One director commented: ‘Government . . . is critical because we are not that well resourced, and have to charge for our services, so government is not a strong advocate. Major critics are people who take a prescribed view of what a systematic review of evidence is.’ Similarly, another director said: ‘Our strongest critics tend to fall into three camps. One group says systematic reviews take too long and cost too much compared to other processes, like expert opinion processes. Other groups say we are too restrictive, and then there is a group that feels we are not restrictive enough.’ Another director noted: ‘Our critics would be those people who would like to use our evidence and can’t afford it.’**

#### Examples of successes and failures

Most of the examples of success among organizations producing CPGs, HTAs, or both were occasions where there was a perception that clinicians adhered to the organization’s recommendations or policymakers based their decisions (at least in part) on the work of the organization. Only one organization producing CPGs, HTAs, or both could not identify an example of success but on the other hand only one organization cited data from an audit to support the perception that clinicians adhered to the organization’s recommendations. In three of the examples of policymakers acting on the work of an organization, an intervention was recommended and policymakers’ subsequent support for the intervention was perceived as a success. **One director who gave several examples said: ‘I think we have good provision of some expensive devices or drugs that are not widely avail[able] in other countries in region.’ Another noted a specific example: ‘We reviewed a new psychiatric drug given to elderly psychiatric patients and supported its supply. It was a good decision because it helped a weak part of the population. It was a drug which has successful results with this group. There is no strong lobby group for these people so it was particularly heartening to see our recommendation get up.’** In another three of the examples of policymakers acting on the work of an organization, an intervention was not recommended and policymakers’ subsequent lack of support for the intervention was perceived as a success. One director cited a Minister’s decision not to start a screening program, and a second cited a Minister’s decision not to fund an expensive new technology, despite lobbying. A third director cited the example of a decision not to fund a drug and argued that this decision had saved lives and money:

**‘It was the whole area of COX-2 inhibitors. About five years ago we attempted to do a systematic review. There was no publicly available evidence. As evidence became available we continued to assess it. We found that their benefits would not justify use. As a result the policymakers made decisions to limit their use. Eventually at least two drugs were taken off the market. Another interesting thing happened. We evaluated utilisation of drugs, so we could compare the impact of that decision. So, two things emerged: One, several outside independent groups looked at this. This jurisdiction, because they decided not to have free use, saved money and in terms of real outcomes, actually a big difference between two jurisdictions. [The one] that followed the recommendations; they had fewer deaths and hospitalisations than [the other] that allowed unrestricted use. It has been published.’**

Two examples of success were drawn from the field of public health: one that addressed smoking cessation, where success was attributed to good timing; the other addressed lowering the legal blood alcohol level for drivers.

The examples of success among GSUs were more diverse and the pathway from research evidence to policy more complex. Several organizations did not identify any examples of success or failure, noting that their role is only to report the research evidence and the decision about whether and how to act on the research evidence is best left to others. The examples of success again tended to represent occasions where policymakers based their decisions (at least in part) on the work of the organization. One director cited examples of savings and improved accessibility to effective drugs from using generic drugs and supporting local producers. Another director cited savings from the discounts that could be negotiated based on drug class reviews. Other domains where success had been achieved included evaluations of a national health reform, healthcare financing policies, implementation of a human resources policy leading to re-categorising health professionals, provision of funds by a donor agency to support local coordination of HIV programs, and a housing policy.

The so-called failures typically involved the perception that clinicians were not adhering to the organization’s recommendations or policymakers were not basing their decisions (at least in part) on the work of the organization. Reasons ranged from insufficient awareness-raising among decision-makers to political lobbying by the patient groups, specialists and companies directly affected by the decision. Often the failures involved a technology not being recommended but policymakers deciding to fund it anyways. However, one failure involved a technology being recommended but not being funded by government. Among the four examples of failures that pertained to broader health system policies, two recommendations were complex and a clear explanation was not offered as to why they were not acted upon (even though one would have saved the government money), one recommendation was likely not acted on because it was too broad, and one (involving cuts to the number of hospitals or to the number of beds within hospitals) was likely not acted on due to political opposition. Several other ‘problems’ were noted as well, such as insufficient research evidence, use of an intervention beyond its recommended uses, and inadequate monitoring of adherence to guidelines through audit. **One director said: ‘We did an assessment of helicopter medical emergency services. In 2000 it was equivocal. In some areas more research is needed. That was interesting. It is being used by both opponents and supporters. It was debated in the press for a year and a half. It will be interesting to see what happens. There is not enough data to say.’ Another director said: ‘Once you have included something in the list, having it available for a particular condition, may involve it being used for other conditions, so the costs blow out.’**

#### Other strengths and weakness

When asked about any other strengths and weaknesses in how the organizations are organized, directors repeated many of the same strengths that were described previously (*e.g.*, independence, particularly from the pharmaceutical industry, close links to decision-makers, well trained and committed staff, use of rigorous methods, an interdisciplinary, collaborative approach, stakeholder involvement, and international collaboration), as well as many of the same weaknesses (*e.g.*, a lack of well trained staff, insufficient resources, inadequate international collaboration, the amount of time, energy and resources required, and unrealistic expectations of clients). The relatively small size of the organizations was viewed by many organizations either as a strength or as both a strength and a weakness. **One director noted: ‘One of the strengths is being small. Everyone works well with each other. There is a good team spirit; we have very good IT [information technology] systems. There are 19 people all up in the group.’ Another noted: ‘A strength is it is small, a weakness is it is small.’ Similarly, a third director noted: ‘The organization’s strength is that it is fairly small and manageable and the staff have different background and skills. Weaknesses include (also) that we are small.’** The relatively small size of the organizations and the relatively low pay of those working in the organizations were viewed by some organizations as a weakness. **One director said: ‘A weakness is that we don't have enough people to do the work. It’s not glamorous, so it’s hard to get people.’ Another noted: ‘We are low paid.’ How the organization is organized was mentioned much less frequently as a strength or weakness. One director noted that: ‘One of the weaknesses is the way the organization is organised. It should be organised into functional teams, rather than on a skills basis. At the moment you’d have all the information officers managed in one team, and not as functional team members supporting the group doing the review. This would be a better way of organising people.’**

#### Advice to others

The advice offered to those trying to establish similar organizations can be grouped into seven main recommendations.

**Collaborate with other organizations**

Most directors emphasised collaboration as important both in establishing an organization and in the ongoing work of an organization. **One director recommended: ‘Go and see how others do it, don’t reinvent the wheel, lots of people are working on this, the methodology is there if you want it.’ Another said: ‘The most important point is to work in collaboration with others . . . nationally and internationally.’ A third director said: ‘It’s important to work together with organizations in different countries because we can share work and design work, particularly if there are topics of common interest . . . you need to be aware that there are a lot of groups doing this sort of work already [internationally]. Find out who they are, and if possible, work with them, rather than duplicate . . . efforts.’**

**Establish strong links with policymakers and involve stakeholders in the work**

Many directors, particularly those working in GSUs, strongly recommended that organizations ‘establish links to policymakers.’ **One suggested: ‘You need a lot of support from the top level of the ministry. Sometimes your conclusions, recommendations or reports will bother interest groups. If not supported by high officials in the ministry, the reports will end up in drawers of your desk.’ The same person went on to describe how they worked with senior policymakers: ‘We first got together with all of them . . . We tried to establish good relations with them. You want them to use your information for change. If you from the beginning close communication channels, your information will end up being irrelevant.’** A number of directors from across all types of organizations also stressed the importance of involving stakeholders. **One suggested: ‘. . . involve clinicians as much as you can.’ Another responded: ‘In our guideline we invited doctors who we felt were treating the very poor . . . Disadvantaged populations should be well represented in the panel.’ A third said that it is ‘important to reign in all stakeholders and make them relevant to policy decision-making.’**

**Be independent and manage conflicts of interest among those involved in the work**

While many directors argued for establishing strong links with policymakers and involving stakeholders in the organization’s work, a number of them highlighted the importance of being independent and managing conflicts of interest. **One director working in a GSU suggested you ‘need an independent organization, not being commanded.’ Another individual, this one working in a organization producing CPGs, HTAs, or both, said: ‘The most important thing is to keep your independence. We learned it’s very difficult to do that, because a lot of people do have strong conflicts of interest if they are accomplished in their field. It becomes a very difficult thing to maintain.’ A director working in the same type of organization noted: ‘Independence seems to be a very import thing and also having very clear conflict of interest guidelines.’ Another said: ‘Address conflicts of interest at the very start of the organizational process, when choosing panellists….’ Yet another offered these comments:**

**‘I’ll give an example, quite a big scandal. WHO reviewed and endorsed hypertension guidelines. They lowered threshold, not based on any reliable evidence. At the time it created quite a stir. As people looked in, it became clear that the groups that developed the guidelines had been funded by drug industry. A lot of people were in a conflict of interest. The people in conflict of interest, their interests had been in increasing utilisation of certain drugs, and clear outcome of guidelines, greatly enlarging the pool of patients that would be diagnosed with that condition.’**

**Build capacity among those working in the organization**

Many directors emphasised the challenge and the importance of recruiting or training multidisciplinary staff. **One director noted: ‘Right from start you need trained people. At least two or three.’ Another commented: ‘I think one of most crucial things is hiring the staff. We have been very lucky with that. Often what we do is employ people on a project basis. We are so dependent on people who work with us, that they are thorough and well-trained, and that they share values of transparency.’ A third suggested ‘you have to train everyone.’** A couple of directors noted the importance of having a multidisciplinary team and, specifically in LMICs, thinking internationally**: ‘We haven’t hesitated in taking researchers from other backgrounds, so we have an international team . . . It also means we can build capacity locally.’** Several directors, particularly those working in GSUs, emphasised the importance of leadership capacity. **One person suggested: ‘You need good leadership and a lot of networking. Without networking it’s not going to work, because you need good leadership. If senior management is good, it trickles down.’ Another recommended that you ‘got to have someone commit his or her lifetime in this arena. There is a need of many researchers and a good understanding of the whole. You need to understand from the grass roots, the stakeholders’ ideology and power play between different constituencies. All this requires a lifetime work in this arena . . . You need to accumulate this understanding and institutionalise this understanding.’**

**Use good methods and be transparent in the work**

Many directors stressed the importance of using good methods and being transparent. **One director recommended: ‘Stick to good methods. That’s the cornerstone. You need to be transparent and evidence-based, and everything else really comes from that. Another director, this one from a high-income country, suggested: ‘Demand excellence in the process of evaluating evidence. Demand excellence and don't ever do anything to prejudice the results. Make sure your processes are fully transparent.’ A director from a middle-income country suggested: ‘The first thing they need to guarantee is information of good quality. In [low- and middle-income] countries . . . you can’t aspire to excellent quality, but you can aspire to good information. If people are going to believe you, you need good information.’ Another director pointed out the importance of clearly defining the role of experts: ‘it is important to define the role of health professionals as experts; *i.e.*, we use health professionals on some aspects of questions, but they don’t make the decisions, but help us to make the decision.’**

**Start small, have a clear audience and scope, and address important questions**

A number of directors stressed the magnitude of the work involved and hence the importance of starting small, having a clear audience and scope, and addressing important questions. **One director noted: ‘. . . it’s endless work, and sometimes quite overwhelming when you look at what needs to be done. Lots of work for sometimes a small return.’ Another said: ‘In general, the work has been well regarded and undertaking it is worth doing, but one shouldn't underestimate the level of work involved.’ A third suggested: ‘Start small, because you can easily be swamped with requests. The other important thing is to have a carefully defined audience (client base) and a carefully defined scope.’ Another suggested: ‘Start slow, start small but be clear of your own scope and collaborative.’ A fifth director noted: ‘. . . you have to be patient . . . and recognize that it is a time-consuming process.’** And while several directors pointed out the need to address important questions, no consistent advice emerged about how to approach the selection of questions. **One director suggested selecting questions that can be answered and to: ‘make it small enough.’ Another suggested: ‘One shouldn’t be frightened off by ‘insufficient evidence’ findings. There is a widespread fear of this. Shining the light where it is currently dark is also worth doing.’**

**Be attentive to implementation considerations even if implementation is not a remit**

Several directors noted the importance of implementation. **One suggested: ‘Be pragmatic and be affiliated in the process of getting yourself heard, by integrating yourself into the process.’ A second said: ‘In general terms, I would want to employ someone with a specific implementation role. A specific dissemination or user-engagement role would be good too.’ A third person recommended: ‘You should pay careful attention to getting reviews implemented. Reviews aren’t good to anyone if results aren’t acted upon.’** A number of directors who did not comment on implementation had made clear that implementation is not part of their organizations’ work; however, some of these directors indicated that implementation considerations still inform their work even if responsibility for implementation lies elsewhere.

#### Roles for WHO

Only a small number of directors provided comments about WHO’s potential role, however, these comments almost always pertained to the role that WHO is or could be playing in fostering collaborations across organizations. **One director noted that ‘ . . . the work that WHO is doing and getting groups talking to each other’ is a step forward. Another noted that: ‘WHO is very important because it commissions and produces work that is relevant to the European context and promotes a form of networking.’ A third said: ‘WHO could provide grants of financial assistance for collaboration between countries. This could improve better health services to larger groups across populations. We exchange a lot of [information] with Canada and we consult each other. If the WHO could help to coordinate and carry out research on important issues on a global basis this would be very beneficial.’ A fourth suggested:**

**‘WHO — we all have something to learn from each other. Everyone has slightly different ways of doing things, implementing policies, so sharing information would be great. If we were to use the same methodology in weighing up evidence, there would be better opportunities to share work. We are disappointed that there are not enough opportunities for collaboration; would like more opportunities for sharing information. Technology is an extensive business, and WHO is uniquely poised to make this technology available to other countries. There must be opportunities for organizations like this one to at least put a portion of our service into helping third world countries. There should be more altruism in this area.’**

**One director envisioned an additional role for WHO: ‘There is a need for WHO to play a more active role. We need to market things to policymakers so that our research can be better used. It would be good if WHO could develop guidelines on this.’**
